# Neuropsychiatric symptoms in a patient with Cushing’s syndrome

**DOI:** 10.4102/sajpsychiatry.v28i0.1706

**Published:** 2022-01-18

**Authors:** Reyna Daya, Faheem Seedat, Emilia Blomerus, Saajidah Bulbulia, Zaheer Bayat

**Affiliations:** 1 Department of Internal Medicine, Division of Endocrinology, Helen Joseph Hospital, Johannesburg, South Africa; 2 Division of Endocrinology and Metabolism, Department of Internal Medicine, Faculty of Health Sciences, University of the Witwatersrand, Johannesburg, South Africa

**Keywords:** Cushing’s syndrome, psychosis, neuro-psychiatric disease, depression, cortisol

## Abstract

Cushing’s syndrome (CS) may present with different neurological and/or psychiatric symptoms including anxiety, depression, cognitive impairment and psychosis. Psychosis is a rare clinical manifestation, with literature limited to case reports. We report a case of a 52-year-old woman with psychosis secondary to CS who was mis-diagnosed as schizophrenia-like psychosis. This case highlights the importance of considering CS as a differential when ruling out medical causes in patients with either new or persistent mental health disturbances.

## Introduction

Cushing’s syndrome (CS) is a collection of signs and symptoms because of prolonged exposure to either endogenous or exogenous excess glucocorticoids (GCs) with disruption to normal hypothalamic-pituitary-adrenal feedback.^1^ Endogenous CS results from chronic excess GC production by the adrenal glands. Cushing’s syndrome is categorised as adrenocorticotropic hormone (ACTH)-dependent (80% – 85%) or ACTH-independent (15% – 20%).^2^ Adrenocorticotropic hormone-dependent CS commonly occurs secondary to a pituitary corticotrophic adenoma (Cushing’s disease), whilst less frequently it may be caused by an extra-pituitary tumour (ectopic ACTH syndrome) or rarely a tumour secreting corticotropin-releasing hormone (CRH).^3^ Excess cortisol production in ACTH-independent CS results from unilateral adrenocortical tumours, which may be benign or malignant. Bilateral macronodular adrenal hyperplasia is a rare cause of ACTH-independent CS.^3^ In 1912, Harvey Cushing was the first to describe psychiatric illness in CS as emotional disturbances.^4,5^ Changes in mental status are a rare initial clinical presentation of CS, although if present are often the first manifestation perceived by family members. Neuro-psychiatric manifestations of CS include sleep disturbances, cognitive abnormalities, depression, anxiety, mania, hypomania, poor short-term memory, poor concentration and psychosis.^2,5^

## Case report

A 52-year-old woman was brought to hospital by her daughter with a 4-day history of manic symptoms, labile mood, insomnia and increased energy levels associated with religious delusions and aggression. She had no physical complaints. She had a past medical history of Type 2 diabetes mellitus (T2DM), treated with metformin; and hypertension (HT) treated with amlodipine, hydrochlorothiazide and atenolol. She had a notable history of multiple prior admissions to psychiatric facilities for psychosis, all within the last 3 years. Prior to this, there was no history of any mental health disorders. Previous psychotropic medications prescribed included lithium, quetiapine, sodium valproate, risperidone and citalopram. On mental state examination (MSE) she was talkative with increased mood and labile affect. She exhibited religious delusions and was intermittently aggressive. Following assessment, she was involuntarily admitted to the psychiatric unit.

Physical examination revealed a typical cushingoid appearance with moon facies, dorso-cervical fat pad (buffalo hump), facial plethora, multiple areas of bruising and central obesity with purple striae. The rest of the clinical examination was normal and she did not have any features to suggest any acute infection. She was referred to the Endocrine unit for further investigation.

Baseline investigations were normal and excluded causes for her confusion. Her glycosylated haemoglobin A1c (HbA_1C_) measured 7.7%. Further work-up revealed an elevated 8 AM cortisol level of 886 nmoL/L (133–370 nmoL/L), with a paired low adrenocorticotrophic hormone (ACTH) level of < 0.2 pmoL/L (1.6–13.9 pmoL/L). She underwent a low-dose (1 mg) dexamethasone suppression test, which failed to suppress (Table 1). A 24-h urine cortisol could not be performed because of the patient’s mental state as she was unable to comply with basic instructions.

**TABLE 1:** Biochemical investigations.

Test	Result	Reference range
Random cortisol	886	133 nmoL/L – 537 nmoL/L
Midnight cortisol	745	<50 nmoL/L
8 AM ACTH	<0.2	1.6 pmoL/L – 3.9 pmoL/L
1 mg dexamethasone suppression test – 8 AM cortisol	720	<50 nmoL/L

ACTH, adrenocorticotropic hormone.

**FIGURE 1:**
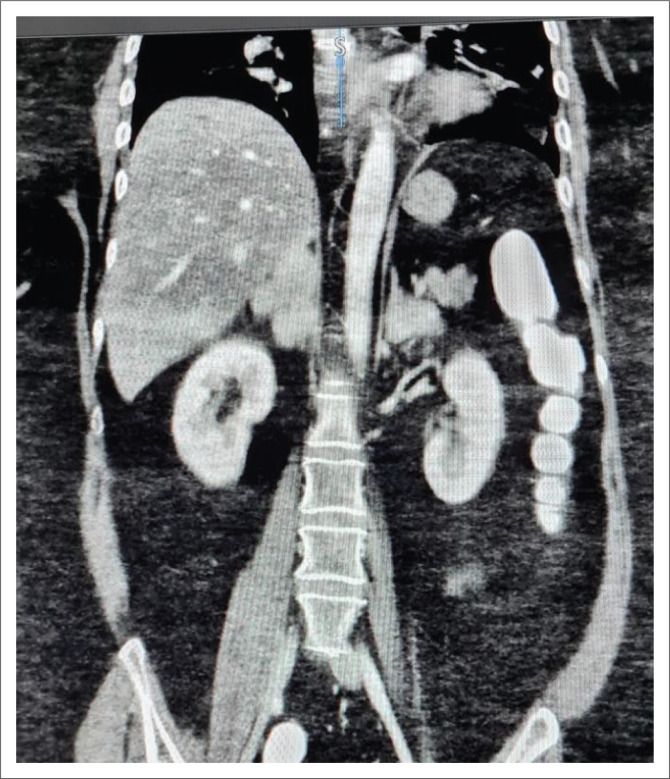
Computed tomography (coronal view) demonstrating bilateral adrenal hyperplasia with the right adrenal gland measuring 44.7 mm × 30.3 mm and the left adrenal gland measuring 25.0 mm × 27.7.mm.

**FIGURE 2:**
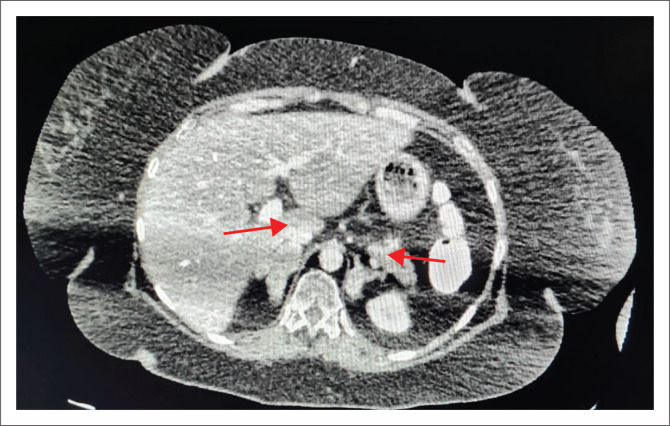
Computed tomography (axial view) demonstrating bilateral adrenal hyperplasia (arrows).

Abdominal contrasted computed tomography (CT) scan showed bilateral lobulated adrenal masses, with the right being larger than the left (right: 44.7 mm × 30.3mm, left: 25.0 mm × 27.7mm) (Figures 1 and 2), containing fat and soft tissue with no calcifications. Absolute washout of radiocontrast after 10 min was 65% bilaterally and the masses measured nine Hounsfield units (HU). Computed tomography Brain showed a normal pituitary gland. Dual-energy X-ray absorptiometry (DXA) scan demonstrated osteoporosis of the left femoral neck (T-score – 3.0).

A diagnosis of CS secondary to bilateral macronodular adrenocortical hyperplasia was made. The CS was complicated by HT, T2DM and osteoporosis and was the likely cause of her psychosis. The patient remained psychotic throughout the admission. Following counselling with the family/legal guardian, a decision for bilateral laparoscopic adrenalectomy was taken.

Peri-operatively, the patient was commenced on intravenous hydrocortisone in order to avoid an adrenal crisis because of the anticipated sudden post-operative reduction in blood cortisol level once the adrenal glands were removed. Post bilateral adrenalectomy she was found to be calm, co-operative, apsychotic and stable. She was able to give a good account of herself and was orientated to time, place and person. Unfortunately, because of subsequent post-operative complications, she demised in the intensive care unit 4 days later.

Histology of the adrenal specimens showed no evidence of invasive malignancy. The increased size, weight and predominant cellular component of the adrenal glands support the diagnosis of bilateral macronodular adrenocortical hyperplasia.

## Discussion

Clinical presentation of CS is variable as patients may exhibit few or all of the signs and symptoms of CS. In addition to HT, T2DM and osteoporosis, patients may present with weight gain, excess hair growth (women), thin skin, moon-shaped round face, fatigue and menstrual irregularity. However, these features are often indiscriminate and may overlap with other diseases such as metabolic syndrome or polycystic ovarian syndrome.^2^ The following five signs have been identified as specific for CS: easy bruising, violaceous striae, proximal myopathy, facial plethora and, in children, weight gain with decreasing growth velocity.^1^ It is important to note that if these features are detected on clinical examination, the patient should be screened for CS.

The relationship between CS and neuro-psychiatric disease is complex. Not only may CS be associated with neuro-psychiatric symptoms, underlying primary psychiatric conditions may increase serum cortisol, resulting in pseudo–CS. All three recommended screening tests used to confirm the biochemical diagnosis of CS (24–h urine free cortisol, midnight salivary cortisol and 1 mg low dose dexamethasone suppression test) may exhibit false positive results in pseudo-CS. To distinguish pseudo-CS from true CS it is recommended that the 48-h 2 mg dexamethasone suppression followed by CRH stimulation is performed.^1,2^

The reported rates of occurrence of neuro-psychiatric symptoms in CS is varied. Psychosis (8%) and mania (3%) are rare^5^ whilst depression is the most common psychiatric disturbance occurring in 50% – 81% of cases.^2,5,6^ Importantly, in up to 12% of cases depressive illness may precede other signs of CS.^2,5,6^ Risk factors for depression in patients with CS include older age, female and the presence of severe hyper-cortisolemia.^7,8^ Memory is the most extensively investigated domain in CS patients and memory impairment is most prominent in active disease.^9,10^ Two-thirds of CS patients report anxiety or panic disorders which occurs during the chronic and advanced stage of disease.^6^ Psychosis is most commonly present in patients with adrenal carcinomas and may be related to the greater degree of hypercortisolemia.^5^

The underlying pathogenesis of neuropsychiatric disease in CS is yet to be fully elucidated. Glucocorticoids receptors are distributed throughout the central nervous system, in particular; the hippocampus, amygdala, limbic system and pre-frontal cortex, rendering these regions particularly vulnerable to hypercortisolemia.^7,9^ A number of theories have been postulated to explain the mechanisms of GC associated neuro-psychiatric disturbances. Firstly, CS is associated with a generalised reduction in cerebral glucose metabolism. Hypercortisolemia is thought to reduce cererbral glucose utilisation resulting in cerebral atrophy. Secondly, GCs increase both the release and effects of neurotoxic excitatory amino acids, such as glutamate, resulting in hippocampal dendritic atrophy. Thirdly, GCs reduce neurotropic factors such as nerve growth factor-b and brain-derived neurotropic factors. Finally, GCs suppress neurogenesis in the dentate gyrus.^6^ All of these hypotheses appear to explain the hippocampal damage responsible for the neurocognitive disorders associated with CS.

Regarding the association of psychosis and CS specifically, it has been postulated that GCs enhance dopaminergic activity with resultant psychosis, which following therapy, explains the resolution of psychosis.^11^ However, much work is needed to clarify the exact mechanisms of neuro-psychiatric disease in patients with CS.

The onset of neuro-psychiatric disturbances in CS is wide ranging.^2^ Psychiatric symptoms might precede, occur during, or even persist following biochemical resolution of CS.^5,12^ A review of the current literature suggests that after correction of hypercortisolism, resolution of psychiatric symptoms is variable. Whilst the treatment and cure of CS significantly improves psychiatric symptoms, there is much debate as to whether complete resolution of neuro-psychiatric symptoms occur following biochemical cure of CS.^6^ Whilst, the majority of patients will improve in the first week, in up to 10% – 45% of patients, full resolution of symptoms is not achieved.^5,12,13^ Despite long-term biochemical remission of CS, recent studies have illustrated persistent deficits in attention, visuo-spatial processing, reasoning, fluency, and short-term memory.^7^

Our patient had psychosis, HT, T2DM and osteoporosis as manifestations of CS. Delirium and other medical causes of psychosis were excluded. There are only a few reported cases of psychosis preceding the diagnosis of CS, as was the case in our patient and to the best of our knowledge there is only one other case reported where psychosis occurred as a primary manifestation of CS because of primary bilateral nodular hyperplasia.^14^ Post-operatively our patient’s mentation and psychotic symptoms resolved but sadly she demised because of post-operative complications.

## Conclusion

Whilst neuro-psychiatric illness is common in patients with CS, it is a rare and unusual initial presenting symptom. A delay in diagnosis may subject a patient to unnecessary psychiatric hospitalisations and psychotropic medications. Clinicians should be aware of this rare entity and have a high index of suspicion to prevent morbidity and mortality associated with CS, especially in the presence of suggestive clinical signs.
